# Composting performance, nitrogen retention, and microbial succession differ among urea-only and organic co-substrate-amended *Camellia oleifera* shell formulations

**DOI:** 10.1186/s40643-026-01161-x

**Published:** 2026-11-12

**Authors:** Kun Sun, Jinping Zhang, Xiaoyan Chu, Hongxia Xu, Benzhi Zhou, Ying Rao

**Affiliations:** 1 Research Institute of Subtropical Forestry, Chinese Academy of Forestry, Hangzhou, 311400 Zhejiang China; 2 Qianjiangyuan Forest Ecosystem Research Station, National Forestry and Grassland Administration of China, Hangzhou, 311400 Zhejiang China; 3 Jiande Forestry Bureau, Jiande, 311600 Zhejiang China; 4 Qiandao Lake Forest Farm, Chunan, 311700 Zhejiang China

**Keywords:** *Camellia oleifera* shell, Aerobic composting, Nitrogen retention, Organic nitrogen sources, Microbial succession, Compost maturity

## Abstract

*Camellia oleifera* shell is a lignocellulosic by-product, and its inefficient decomposition limits waste reduction and nutrient recovery. To identify practical amendment strategies, we compared urea alone (CK) with urea combined with goat dung (GD), *C. oleifera* seed meal (CM), or rice bran (RB) in terms of nitrogen retention, compost maturation, and microbial succession during *C. oleifera* shell composting over 77 days. Organic co-amendments improved compost heating and prolonged thermophilic conditions relative to urea alone. RB achieved the highest nitrogen retention (91.64 ± 0.96%), whereas CK had the lowest measured inorganic nitrogen concentration. Co-amendments increased bacterial diversity and were associated with succession of fungal communities toward saprotrophy. Bacterial community patterns were related to the carbon/nitrogen (C/N) ratio, electrical conductivity (EC), and the extract optical index (E4/E6), while fungal succession was more strongly associated with composting time. Overall, supplementing urea with organic co-substrate was associated with nitrogen retention and several maturity indices relative to urea alone, although no single amendment performed best on every compost quality criterion. Although RB’s final total nutrient content was below the cited 4.0% criterion and final-product safety was not established, these findings provide a practical basis for converting *C. oleifera* shell into a more stable, nutrient-retaining compost and support the resource-efficient utilization of this agricultural by-product.

**Graphical abstract:**

**Figure:**
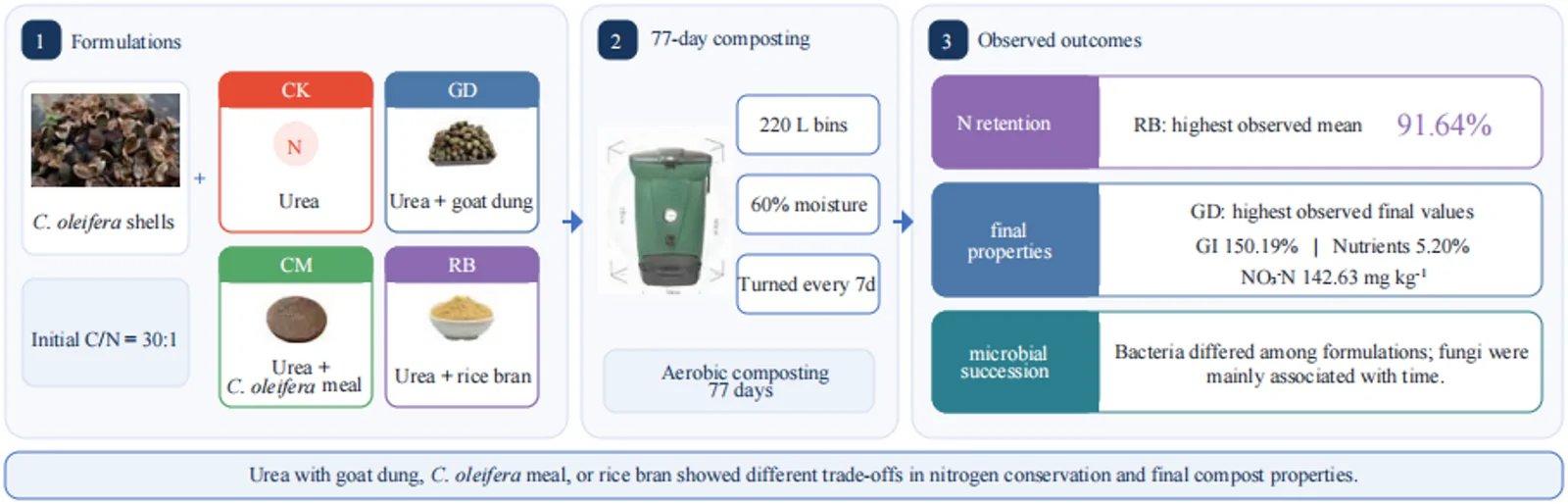

**Supplementary Information:**

The online version contains supplementary material available at https://doi.org/10.1186/s40643-026-01161-x.

## Introduction

*Camellia oleifera* shell is a lignocellulosic by-product generated during the processing of *C. oleifera*: approximately 0.54 t of shell is generated per tonne of processed fruit (Zhang et al. 2021). An earlier estimate placed annual shell production in China at 3.6 million tonnes in 2017 (Zhang et al. 2020a), illustrating the scale of the resource without assuming that the same figure applies to the present year. Shells are often discarded or burned, while other valorization routes include extraction of chemicals, manufacture of bio-based materials, and pyrolysis (Zhang et al. 2020a, 2021). Composting offers a practical route when the intended product is a soil amendment or cultivation substrate: it can process shell together with locally available organic residues, return organic matter and nutrients to land, and avoid leaving the material untreated (Zhang et al. 2021; Liu et al. 2025). These are application-specific advantages; a general superiority over extraction or pyrolysis cannot be inferred without a comparative life-cycle and economic assessment.

However, *C. oleifera* shell has a high carbon/nitrogen (C/N) ratio and is rich in recalcitrant lignocellulose, tannins, saponins, and related compounds. These properties limit the nitrogen available for microbial assimilation and often lead to slow temperature rise, low degradation efficiency, and an extended maturation period (Liang et al. 2024). Managing the composting process using external additives has therefore become an important means of improving the efficiency of *C. oleifera* shell composting.

Our previous work showed that different additives can substantially alter the chemical composition and microbial diversity of *C. oleifera* shell during composting. In particular, co-composting materials such as *C. oleifera* seed meal (CM), *C. oleifera* seed cake, and goat dung (GD) were found to affect composting progress and bacterial community structure (Zhang et al. 2021). Because those treatments contained organic co-substrates and emphasized composting performance and community diversity, they did not establish how formulations containing different organic co-substrates compare with urea alone for nitrogen retention at the same initial C/N ratio. The present study addresses that specific formulation-level question while examining bacterial and fungal succession.

Urea is convenient for adjusting the initial C/N ratio, but it is rapidly hydrolyzed to form NH_4_^+^–N. When the release of NH_4_^+^–N exceeds the capacity of microbial assimilation, nitrogen can be lost through NH_3_ volatilization, particularly under thermophilic and weakly alkaline conditions (Jiang et al. 2011). Nitrogen can also leave a composting system as nitrous oxide (N_2_O) or dinitrogen (N_2_) following nitrification and denitrification, and in leachate if water is not adequately controlled (Sun et al. 2025). Enhanced microbial assimilation of ammonium into biomass is one retention pathway (Mo et al. 2023; Liang et al. 2024). Other approaches include matching nitrogen release to microbial demand through feedstock choice and C/N adjustment; controlling aeration, moisture, and pH to limit NH_3_ volatilization and anaerobic denitrification; retaining NH_4_^+^ by sorption or chemical immobilization; and regulating nitrification–denitrification so that conversion to nitrate does not merely shift losses to N_2_O or N_2_ (Yang et al. 2023; Sun et al. 2025). Biochar and mineral amendments can reduce some gaseous losses under particular conditions, but their effects depend on the feedstock and process (Yang et al. 2023). Changes in nitrogen species or microbial composition alone do not identify a dominant pathway without direct measurements of gaseous and leachate losses.

In this study, GD, CM, and rice bran (RB) were selected as three representative organic co-substrates. GD represents a livestock-manure-derived organic nitrogen system; co-composting GD with high-carbon materials such as straw can improve pile structure and support the maturation process (Kulcu and Yaldiz 2007). CM and *C. oleifera* shell originate from the same processing chain and therefore represent the internal recycling of by-products from *C. oleifera* processing. During co-composting of *C. oleifera* shell and seed cake, tannins and saponins are degraded alongside changes in nutrient composition (Zhang et al. 2020a, b). RB represents a grain-processing by-product; because RB is rich in carbohydrates, proteins, and minerals, it can provide a profile of readily available carbon and organic nitrogen release that differs from those of GD and CM (Saunders 1985). Comparing these three organic co-substrates in combination with urea under the same initial C/N ratio can therefore help clarify whether the effects of nitrogen sources depend on co-substrate origin.

Microbial succession is a key link between nitrogen-source composition and nitrogen retention. Bacterial communities change markedly across composting stages (Partanen et al. 2010), and feedstock origin can influence the abundance of functional genes related to nitrogen transformation, thereby altering the extent of nitrogen loss (Yin et al. 2022). In composting systems based on nitrogen-containing organic wastes, shifts in microbial communities and nitrogen-transformation functional genes can jointly affect ammonification, nitrification, and denitrification (Yang et al. 2019). In addition, interventions that improve nitrogen retention are often accompanied by microbial community restructuring and enhanced ammonia assimilation (Li et al. 2022). To move beyond the earlier observation that additives improve composting performance, it is therefore necessary to integrate nitrogen mass balance, nitrogen-species dynamics, and microbial succession throughout the entire composting process.

Accordingly, we compared a urea-only formulation with formulations amended with GD, CM, or RB, all adjusted to the same initial C/N ratio and managed under identical composting conditions. We aimed to characterize differences in composting performance, carbon and nitrogen retention, inorganic nitrogen dynamics, and bacterial and fungal succession among these formulations. We further examined associations between measured physicochemical variables and microbial community composition. We hypothesized that the three organic co-substrates, owing to their combined chemical, physical, and biological properties, would generate distinct composting trajectories relative to the urea-only formulation. This design was not intended to isolate the independent effect of nitrogen form or nitrogen-release rate.

## Materials and methods

### Materials

*C. oleifera* shells, *C. oleifera* seed meal, and fresh goat dung were collected from Chun’an County, Hangzhou, Zhejiang Province, China. Here, “GD” refers to solid goat excrement without bedding materials or litter, rather than mixed GD. CM is the solid residue remaining after the oil extraction from *C. oleifera* seeds. RB was purchased from Lǜwan Agricultural Products Co., Ltd. (Hebei, China), and urea was obtained from Jinkai Chemical Investment Holding Group (Henan, China). The basic physicochemical properties of the raw materials are shown in Table 1. Lignin content and the lignin–to–nitrogen ratio was included because lignin–rich substrates can constrain organic matter decomposition and influence humification–related indicators during composting.

**Table 1:** Basic physicochemical properties of the raw materials used in composting (mean ± SD, *n* = 3)

Raw material	C. oleifera shell	C. oleifera seed meal	Urea	GD	RB
TC (%)	48.60 ± 1.51	47.80 ± 1.42	20	36.50 ± 1.01	43.80 ± 1.73
TN (%)	0.42 ± 0.03	1.22 ± 0.05	46.4	2.78 ± 0.07	2.93 ± 0.06
TP (%)	0.02 ± 0.00	0.16 ± 0.02	NA	0.99 ± 0.10	1.59 ± 0.16
TK (%)	0.85 ± 0.09	0.93 ± 0.09	NA	2.15 ± 0.22	0.48 ± 0.04
C/N ratio	115.71 ± 6.13	39.18 ± 2.42	0.43	13.13 ± 0.81	14.95 ± 1.32
Lignin (%)	29.71 ± 5.04	8.54 ± 0.58	NA	12.33 ± 0.82	7.06 ± 0.50
Lignin/N ratio	70.74 ± 6.21	7.00 ± 0.46	NA	4.44 ± 0.35	2.41 ± 0.08

NA, Not applicable because urea does not structurally contain phosphorus, potassium, or lignin. C/N_*i*_ =TC_*i*_ /TN_*i*_, Lignin/N_*i*_ =Lignin_*i*_ /TN_i_ where *i* = 1, 2, 3. First, calculate the C/N and Lignin/N ratios for each replicate; then, calculate the mean and SD of the three ratios. TC, Total carbon; TN, Total nitrogen; TP, Total phosphorus; TK, Total potassium; SD, Standard deviation; GD, Goat dung; CM, *C. oleifera* seed meal; RB, Rice bran

A commercial microbial inoculant was supplied by Yijiayi Biotechnology Co., Ltd. (Zhengzhou, China). According to the product specification, the inoculant contained Bacillus, lactic acid bacteria, Bifidobacterium, yeast, photosynthetic bacteria, and actinomycetes, with a total viable count of ≥ 2.0 × 10^10^ CFU g^− 1^. Prior to use, the powdered inoculant was activated and prepared as a microbial inoculum solution according to the supplier’s instructions. Briefly, 0.50 kg of brown sugar was dissolved in 5.00 kg of sterilized water and cooled to room temperature. Subsequently, 0.50 kg of the powdered microbial starter was added, and the resulting suspension was sealed and fermented for 12 h. In addition, heavy metal concentrations in the raw *C. oleifera* shell were determined to evaluate potential input risks from the composting substrate. The detailed values are provided in Table S1.

### Experimental design and composting procedure

Composting experiments were conducted at the Research Institute of Subtropical Forestry, Chinese Academy of Forestry, located in the Fuyang District of Hangzhou, Zhejiang Province, China, from February to April 2024. Four treatments were established: one control group with urea only and three treatments combining high-nitrogen organic material with urea, each featuring three independent replicates. In the combined treatments, *C. oleifera* shells were mixed with GD, CM, or RB at a dry weight ratio of 4:1. The CK consisted solely of *C. oleifera* shells supplemented with urea. All treatments were adjusted to an identical initial C/N ratio of 30:1 using urea (Zhang et al. 2020c, 2021).

Composting was performed in 220 L insulated composting bins (BIOLAN, Finland). Before composting, *C. oleifera* shells were crushed to a size of ≤ 10 mm, while *C. oleifera* seed meal and RB were sieved to ≤ 3 mm. The initial moisture content was adjusted to 60% on a wet-weight basis. At each weekly turning event, each compost bin was weighed, the moisture content was measured, and water was added as needed to restore the moisture content to 60%. The activated microbial inoculum solution described in Sect. 2.1 was uniformly mixed into the composting materials at a volume of 1000 mL per bin, measured using a 1000 mL beaker. The same volume was applied to each bin across all four treatments. The initial wet and dry masses of the materials added to each bin, including the treatment-specific urea amounts, are provided in Table S2. The compost piles were manually turned every 7 days. Temperatures at the top, middle, and bottom layers were measured daily at 15:00 using a DTSW–2 digital electronic thermometer, with the average recorded. Each composting bin was regarded as an independent experimental unit. Three independent bins were established for each treatment, giving a total of 12 experimental units. At the beginning of composting, all bins were placed in the same open area and randomly arranged in a 2 × 6 layout, with a spacing of 1 m between adjacent rows and columns. After each turning event, conducted at 7-day intervals, the bins were re-randomized and rearranged in the same 2 × 6 layout within the experimental area to minimize potential positional effects. Approximately 500 g of compost was collected from each bin using a five–point sampling method on days 0, 21, 42, 63, and 77. The mass of material removed at each sampling event was recorded and accounted for in the carbon and nitrogen mass-balance calculations. Each sample was divided into two parts: one was stored at 4 °C for physicochemical analysis, while the other was frozen at − 80 °C for microbial community analysis.

### Physicochemical analyses

Compost samples were air–dried and mixed with distilled water at a ratio of 1:10 (w/v). The mixtures were shaken for 5 min and allowed to stand at room temperature for 30 min. The pH and electrical conductivity (EC) of the extract were measured using a PB–10 pH meter and a DDS-307 A EC meter, respectively (Shanghai INESA Scientific Instruments Co., Ltd., China), following the methodology applied for *C. oleifera* matrix stabilization (Zhang et al. 2021). The concentrations of NH_4_^+^-N and NO_3_^−^-N were determined using standard mineral nitrogen colorimetric protocols (Meng et al. 2018a, b). Total carbon (TC), total nitrogen (TN), total phosphorus (TP), and total potassium (TK) were analyzed to assess nutrient dynamics during composting. Total nutrient content was calculated as the sum of total N, P_2_O_5_, and K_2_O contents, with total P and total K converted to P_2_O_5_ and K_2_O using standard conversion factors (Ministry of Agriculture and Rural Affairs of the People’s Republic of China 2021). The E4/E6 ratio was defined as the ratio of the absorbance of a compost extract measured at 465 nm (E4) to that at 665 nm (E6). Briefly, compost samples were extracted with 0.05 mol/L sodium bicarbonate solution at a ratio of 1:10 (w/v) at room temperature for 24 h, followed by filtration. The filtrate was diluted 100-fold with 0.05 mol L^−1^ sodium bicarbonate solution, and its absorbance at 465 nm and 665 nm was measured using a UV-Vis spectrophotometer (TU-1810DS, China) to calculate the E4/E6 ratio. A 0.05 mol L^−1^ sodium bicarbonate solution was used as the spectrophotometric blank.

The seed germination index (GI) was assessed with 20 seeds of Pak Choi (*Brassica chinensis*) by measuring seed germination and radicle elongation (Bernal et al. 2009). Briefly, compost samples were mixed with deionized water at a solid-to-liquid ratio of 1:10 (g: mL) and shaken at room temperature for 2 h. After centrifugation and filtration, 5 mL of the resulting supernatant was added to a Petri dish lined with filter paper. Twenty seeds were evenly placed in each dish and incubated in the dark at 25 ± 2 °C for 48 h. Seed germination rate and root length were subsequently measured. Deionized water was used as the blank control. The calculation formula is as follows:

$$\:GI\left(\%\right)=\frac{{R}_{1}\times\:{L}_{1}}{{R}_{2}\times\:{L}_{2}}\times\:100$$

Where $\:{R}_{1}$ and $\:{L}_{1}$ are the germination rate (%) and root length (mm) in the compost extract, and $\:{R}_{2}$ and $\:{L}_{2}$ are the corresponding values in distilled water.

Carbon and nitrogen losses were estimated using mass-balance equations based on changes in TC, TN, and dry matter according to Tiquia et al. (2002). Sampling removes carbon and nitrogen from the bins and must be distinguished from process losses. For a sampling-corrected balance, the relevant quantities are as follows:

$$ \begin{aligned} & Carbon\:Loss\:at\:time\:t\:\left( \% \right) \\ & \quad = \left[ {1 - \frac{{DM_{{\mathrm{t}}} \times \:TC_{{\mathrm{t}}} + \Sigma \:{\mathrm{jDs}}\left( {\mathrm{j}} \right)\: \times \:\:{\mathrm{cC}}\left( {\mathrm{j}} \right)}}{{DM_{0} \times \:TC_{0} }}} \right] \times \:100 \\ \end{aligned} $$

$$ \begin{aligned} & Nitrogen\:Loss\:at\:time\:t\:\left( \% \right) \\ & \quad = \left[ {1 - \frac{{DM_{{\mathrm{t}}} \times \:TN_{{{\mathrm{t}} + }} + \Sigma \:{\mathrm{jDs}}\left( {\mathrm{j}} \right)\: \times \:\:{\mathrm{cN}}\left( {\mathrm{j}} \right)}}{{DM_{0} \times \:TN_{0} }}} \right] \times \:100 \\ \end{aligned} $$

where $\:D{M}_{0}$, $\:T{C}_{0}$, and $\:T{N}_{0}$represent the initial total dry-matter mass and the initial TC and TN concentrations, respectively, whereas $\:D{M}_{t}$, $\:T{C}_{t}$, and $\:T{N}_{t}$represent the corresponding values at sampling time * t*. Ds is the corresponding dry-matter-based element mass fraction; and Ds(j) is the dry mass removed at sampling event j. Dry mass is calculated as wet mass × (1 − moisture mass fraction), with bin (or container for samples) tare excluded. Carbon and nitrogen retention at each sampling time were calculated as 100% minus the corresponding carbon and nitrogen loss percentages, respectively. In the formulas, the unit for dry weight is kg. The units for the content of the original substances are all kg/kg.

### DNA extraction, PCR amplification, and sequencing

Total genomic DNA was extracted from 0.25 g of compost samples using the PowerSoil^®^ DNA Isolation Kit (MO BIO, USA, Cat. No. 12888) according to the manufacturer’s instructions. DNA concentration and purity were evaluated by 1% agarose gel electrophoresis, and the extracted DNA was diluted to 1 ng µL^− 1^ with sterile water before PCR amplification.

The bacterial V3-V4 region of the 16 S rRNA gene was amplified using primers 341 F (5′-CCTACGGGNGGCWGCAG-3′) and 805R (5′-GACTACHVGGGTATCTAATCC-3′), yielding an amplicon of approximately 460 bp. The fungal internal transcribed spacer 1(ITS1) region was amplified using primers the barcode-tagged primers ITS1 (5′-TCCGTAGGTGAACCTGCGG-3′) and ITS2 (5′-GCTGCGTTCTTCATCGATGC-3′). PCR amplification was performed using Phusion^®^ High-Fidelity PCR Master Mix (New England Biolabs) according to the sequencing provider’s protocol. PCR products were mixed with an equal volume of 1× loading buffer containing SYBR Green and examined by 2% agarose gel electrophoresis. Products showing a bright main band at the expected size were selected for subsequent analysis. The selected PCR products were pooled in equidensity ratios and purified using the Qiagen Gel Extraction Kit (Qiagen, Germany).

Sequencing libraries were prepared from the purified PCR products using the TruSeq^®^ DNA PCR-Free Sample Preparation Kit (Illumina, USA) according to the manufacturer’s recommendations, and index codes were added. Library quality was assessed using a Qubit 2.0 Fluorometer (Thermo Scientific) and an Agilent Bioanalyzer 2100 system. The libraries were subsequently sequenced on an Illumina MiSeq platform to generate 300-bp paired-end reads. The raw bacterial 16 S rRNA gene and fungal ITS amplicon sequences have been deposited in the National Center for Biotechnology Information (NCBI) Sequence Read Archive under BioProject accession PRJNA1537769 and SRA Study accession SRP741655.

### Bioinformatics and statistical analysis

Paired-end reads were assigned to samples based on their unique barcodes, and barcode and primer sequences were removed. The paired–end reads were merged using FLASH v1.2.11 to generate raw tags. Quality filtering of raw tags was conducted using QIIME v1.9.1 to obtain high–quality clean tags. Chimeric sequences were identified and removed by comparison with the gold reference database using UCHIME, resulting in effective tags. After merging, quality filtering, and chimera removal, 46,897 − 50,559 bacterial sequences and 30,924 − 34,523 fungal sequences were retained per sample.

Sequence analysis was performed using UPARSE v8.1.1861. Sequences with ≥ 97% identity were clustered into operational taxonomic units (OTUs), and representative sequences were selected for taxonomic annotation. An OTU-based pipeline was utilized because the sequencing data were initially processed using the QIIME1/UPARSE workflow. Bacterial OTU representative sequences were compared with SILVA v123 using UCLUST at a 90% sequence-similarity threshold for database matching; this value denotes sequence similarity, not classifier confidence, and should not be interpreted as a reliable genus-level identification threshold. Fungal representative sequences were annotated against the UNITE database public release 01.12.2017 utilizing BLAST with an E–value threshold of 0.001. Multiple sequence alignment was conducted using MUSCLE v3.8.31. Bacterial and fungal OTU abundance tables were normalized separately using the minimum sequence count within each dataset before downstream alpha and beta diversity analyses.

Alpha diversity indices, including observed species, Chao1, Shannon, Simpson, Ace, and Good’s coverage, were calculated using QIIME v1.9.1 and visualized in R v4.5.3. The sequencing dataset contained one pooled microbial profile per treatment-time combination. Alpha diversity indices were therefore interpreted descriptively, without biological-replicate standard errors or significance tests. Beta diversity was assessed using weighted and unweighted UniFrac distances with QIIME v1.9.1. Principal coordinate analysis (PCoA) was conducted using the weighted or unweighted UniFrac distance matrices and visualized using the ggplot2 package in R v4.5.3. UPGMA clustering was performed with QIIME v1.9.1 to examine sample clustering patterns. PERMANOVA was used to preliminary test differences in bacterial and fungal community composition among treatments and composting stages. Ordination separation was not interpreted as evidence of a replicated treatment effect.

Each compost bin was considered an independent experimental unit (four treatments × three bins). Because each bin was sampled repeatedly at five time points, physicochemical variables were analyzed using linear mixed-effects models with treatment, sampling time (categorical), and treatment × time as fixed effects and bin identity as a random intercept. Omnibus P values were adjusted using the Benjamini–Hochberg procedure separately across the eight physicochemical endpoints for each effect family. When the omnibus model supported treatment differences, estimated marginal means were compared among treatments within each sampling time using Tukey-adjusted contrasts.

Bacterial functional profiles were inferred from 16 S rRNA data using Tax4Fun v0.3.1 and the SILVA reference database (Aßhauer et al. 2015). Fungal trophic modes and guilds were assigned using FUNGuildR v0.3.0 based on the ITS OTU abundance table and the corresponding taxonomic annotations (Nguyen et al. 2016). The available annotation dataset contained 726 unique OTUs. Of these, 561 OTUs (77.27%) were retained for functional summaries, including 443 assigned as “Probable” and 118 as “Highly Probable”. The remaining 71 OTUs assigned as “Possible” and 94 unassigned OTUs were excluded. Vertical-bar symbols were removed from guild labels before aggregation, and OTUs with identical resulting labels were grouped together, yielding 71 guild categories. Multi-guild annotations were retained as composite categories rather than split among individual guilds, so each OTU contributed its read count only once. Within each sample, guild relative abundance was calculated by dividing the summed read count of each category by the total read count of all retained OTUs. The 10 categories with the highest cumulative read counts across the 20 samples were displayed separately, and the remaining 61 categories were grouped as “Others”. These assignments represent predicted ecological guilds rather than direct measurements of fungal activity.

Redundancy analysis (RDA) was performed using the vegan package in R to describe associations between measured physicochemical variables and microbial community composition. Bacterial and fungal OTU abundance matrices were analyzed separately following Hellinger transformation, and the environmental variables were matched with the corresponding microbial samples prior to ordination analysis. Fitted environmental vectors were employed to describe the physicochemical variables most strongly associated with variations in the bacterial and fungal communities. To further quantify the relative contribution of each environmental variable to the explained variation in microbial community composition, hierarchical partitioning was performed using the rdacca.hp package in R. This analysis decomposed the total adjusted explanatory power of the RDA model into the independent contribution of each environmental variable (Lai et al. 2022). PERMANOVA was performed on Bray–Curtis dissimilarities calculated from Hellinger-transformed OTU profiles. The previously reported PERMANOVA scheme permuted profiles within sampling times for treatment and within treatment trajectories for time. The former does not preserve each longitudinal trajectory as an intact unit, and the latter requires temporal exchangeability that cannot be assumed for ordered composting observations. Neither restriction recovers the biological replication lost through pooling. Accordingly, these permutation *P* values were not retained as confirmatory evidence. A treatment × time interaction cannot be separated from residual variation with one profile per treatment-time cell. Multivariate dispersion was not assessed in the available analysis; thus, differences in within-group dispersion cannot be excluded as an explanation for apparent community separation.

Within-treatment associations between genus-level relative abundance and physicochemical variables were evaluated using repeated-measures correlation, with treatment specified as the repeated-measures subject. Twenty treatment-by-time profiles (four treatments × five sampling times) were analyzed; replicate physicochemical measurements were averaged within each treatment-time combination before matching to the corresponding microbial profile. Benjamini–Hochberg false-discovery-rate correction was applied separately to the 100 bacterial and 100 fungal genus–environment tests, and adjusted *P* values are reported as q values.

## Results

### Temperature dynamics and carbon and nitrogen retention

**Fig. 1:**
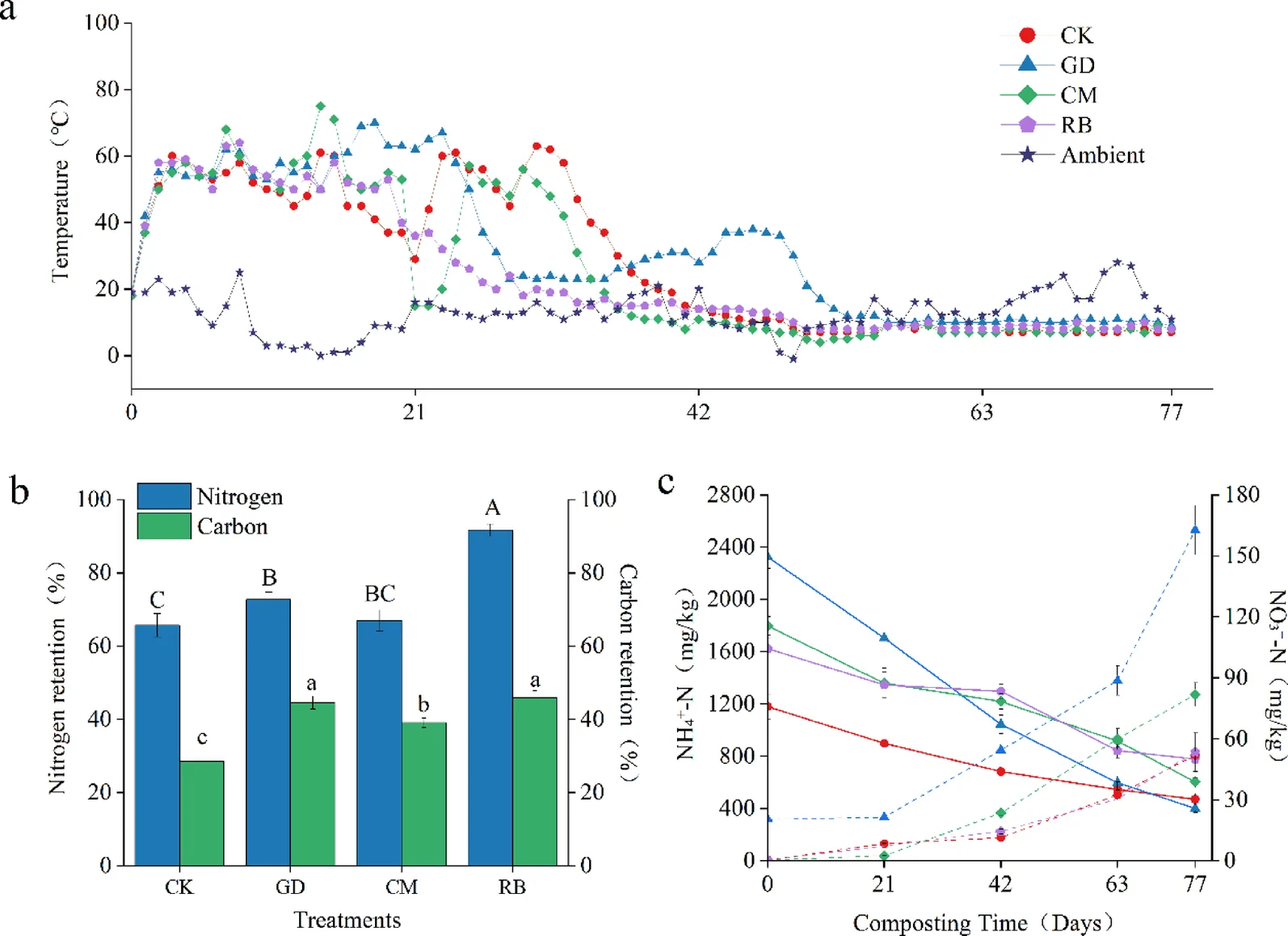
Temporal physicochemical changes during *C. oleifera* shell composting under different nitrogen-source treatments. **A** temperature; **B** carbon and nitrogen retention, calculated using the mass-balance equations described in Sect. 2.3; **C** temporal concentrations of NH_4_^+^-N and NO_3_^−^-N. Solid lines represent NH_4_^+^-N, while dashed lines denote NO_3_^−^-N. *Note:* Error bars indicate ± SE (*n* = 3). Different uppercase (nitrogen) and lowercase (carbon) letters indicate significant differences among treatments at *P* < 0.05. CK: *C. oleifera* shell + urea; CM: *C. oleifera* shell + urea + *C. oleifera* seed meal; RB: *C. oleifera* shell + urea + rice bran; GD: *C. oleifera* shell + urea + goat dung. Abbreviations: SE: standard error

The temperature dynamics of each compost pile are shown in Fig. 1a. All treatments entered the thermophilic stage on day 2, and the overall trend was an initial increase followed by a decline. However, the peak temperatures and their timing differed among treatments. The cumulative thermophilic duration, defined as the total number of days with recorded compost temperatures ≥ 50 °C, including non-consecutive thermophilic episodes, was 20 days for CK, 24 days for GD, 24 days for CM, and 18 days for RB. These results indicate that, under the same initial C/N ratio, different additives could affect the composting process of *C. oleifera* shell differently. After composting, nitrogen retention differed significantly among treatments. The RB treatment (91.64 ± 0.96%) showed significantly higher nitrogen retention than the other three treatments. The GD treatment (72.71 ± 1.18%) was significantly higher than CK (65.65 ± 1.83%) but did not differ significantly from CM (66.97 ± 1.65%). No significant difference was detected between CM and CK. For carbon retention, the RB (45.83 ± 1.17%) and GD (44.56 ± 1.01%) treatments showed significantly higher values than CM (39.03 ± 0.74%), which in turn was significantly higher than CK (28.56 ± 1.36%) (Fig. 1b). These results indicate that the mixed-amendment treatments improved carbon retention relative to urea alone, whereas their effects on nitrogen retention were amendment-dependent, with the strongest improvement observed in the RB treatment.

### Nitrogen transformation

The dynamics of ammonium nitrogen (NH_4_^+^–N) and nitrate nitrogen (NO_3_^−^–N) during composting are shown in Fig. 1c. At the beginning (day 0), NH_4_^+^–N contents followed the order GD group (2324.13 ± 88.03 mg/kg) > CM group (1797.35 ± 71.03 mg/kg) > RB group (1623.94 ± 81.05 mg/kg) > CK group (1178.87 ± 94.14 mg/kg), whereas NO_3_^−^–N contents followed GD group (0.36 ± 0.01 mg/kg) = CM group (0.36 ± 0.01 mg/kg) > CK group (0.32 ± 0.01 mg/kg) > RB group (0.20 ± 0.01 mg/kg). The concurrent decrease in NH_4_^+^–N and increase in NO_3_^−^–N is consistent with nitrification, although nitrifier abundance, nitrification-gene copy number, and enzyme activity were not directly measured, so this interpretation should be regarded as an inference from concentration dynamics rather than a directly demonstrated process. Accordingly, NH_4_^+^-N contents decreased gradually in all treatments, reaching RB group (777.92 ± 46.10 mg/kg) > CM group (603.87 ± 34.06 mg/kg) > CK group (470.71 ± 43.03 mg/kg) > GD group (401.61 ± 32.09 mg/kg) at the end (day 77). Meanwhile, NO_3_^−^–N contents increased gradually, reaching GD group (142.63 ± 12.10 mg/kg) > CM group (81.80 ± 5.90 mg/kg) > RB group (53.45 ± 9.50 mg/kg) > CK group (51.93 ± 4.80 mg/kg) at day 77. The sum of measured NH_4_^+^–N and NO_3_^−^–N was lower in CK group than in the other treatments at both the beginning and the end of composting. Those results suggest that at the same initial C/N ratio, differences in NH_4_^+^–N and NO_3_^−^–N concentrations between the urea-only treatment and treatments combining urea with other materials may reflect variations in inorganic nitrogen transformation, although nitrification rates were not directly measured.

### Compost maturation and quality characteristics

**Fig. 2:**
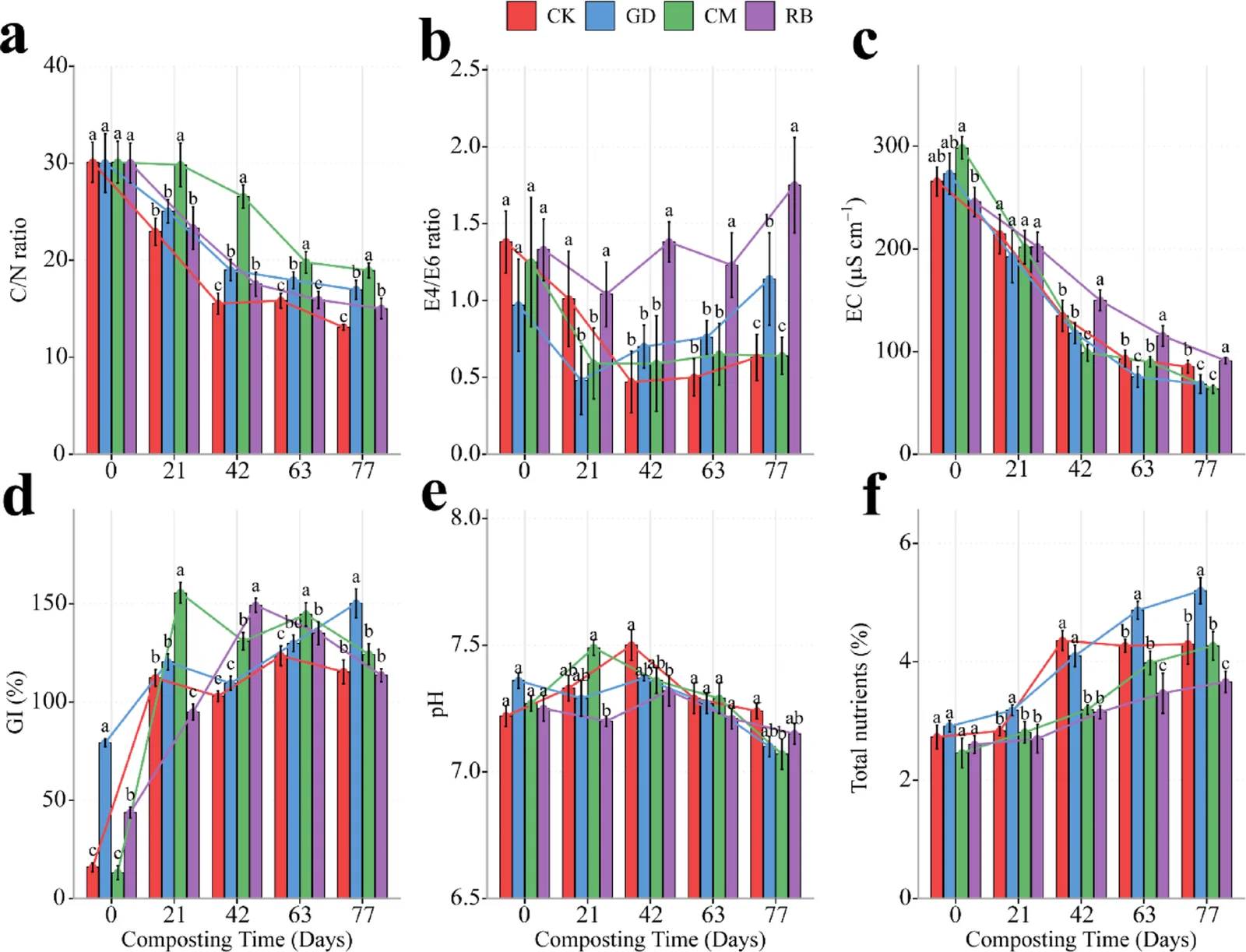
Temporal dynamics of maturity-related parameters during composting under different nitrogen-source treatments. **A** C/N; **B** E4/E6; **C** EC; **D** GI; **E** pH; **F** Total nutrients. *Note:* Error bars indicate ± SE (*n* = 3). Total nutrients include N, P_2_O_5_, and K_2_O. CK: *C. oleifera* shell + urea; CM: *C. oleifera* shell + urea + *C. oleifera* seed meal; RB: *C. oleifera* shell + urea + rice bran; GD: *C. oleifera* shell + urea + goat dung. Abbreviations: C/N: carbon/nitrogen; E4/E6: ratio of absorbance at 465 nm to that at 665 nm in the same compost extract; EC: electrical conductivity; GI: germination index; SE: standard error

The C/N ratio decreased with composting time in all treatments. The reduction was greatest in CK, and its final value was lower than those of the other three groups. The final C/N ratios followed CK group (13.09 ± 0.29) < RB group (15.02 ± 1.04) < GD group (16.95 ± 0.99) < CM group (18.95 ± 0.77) (Fig. 2a). The reported optical index generally decreased first and then increased, although the patterns differed among treatments. In CK group, it declined continuously from day 0 to 42 and rose slightly thereafter. In GD and CM groups, it decreased from day 0 to 21 and then increased. In RB group, it decreased during days 0–21 and 42–63 but increased during days 21–42 and 63–77. The final values followed CK group (0.63 ± 0.15) < CM group (0.64 ± 0.12) < GD group (1.14 ± 0.30) < RB group (1.75 ± 0.31) (Fig. 2b). The EC values decreased over time in all treatments, with final values of CM (63.40 ± 4.06 µS cm^− 1^) < GD group (68.40 ± 9.09 µS cm^− 1^) < CK group (85.31 ± 6.11 µS cm^− 1^) < RB group (91.26 ± 3.03 µS cm^− 1^) (Fig. 2c). The GI values of all treatments increased after day 21 and remained at relatively high levels despite subsequent fluctuations (Fig. 2d). The pH was slightly alkaline at the beginning, rose slightly, and then declined after day 42, with final values of CM group (7.07 ± 0.06) < GD group (7.10 ± 0.04) < RB group (7.15 ± 0.04) < CK group (7.24 ± 0.03) (Fig. 2e). Total nutrient contents increased with composting time in all treatments, with final values of RB group (3.66 ± 0.18%) < CM group (4.27 ± 0.24%) < CK group (4.30 ± 0.34%) < GD group (5.20 ± 0.22%) (Fig. 2f). RB therefore had the highest estimated nitrogen retention but did not meet the cited 4.0% total-nutrient criterion; this comparison does not establish overall compliance or agricultural-use safety. Linear mixed-effects models showed significant treatment × time interactions for the pH, EC, GI, E4/E6, C/N, total nutrients, TN, TC (FDR-adjusted *P* < 0.05; Table S3), indicating that the effects of the amendments varied across composting stages.

### Structure of bacterial and fungal diversity

**Fig. 3:**
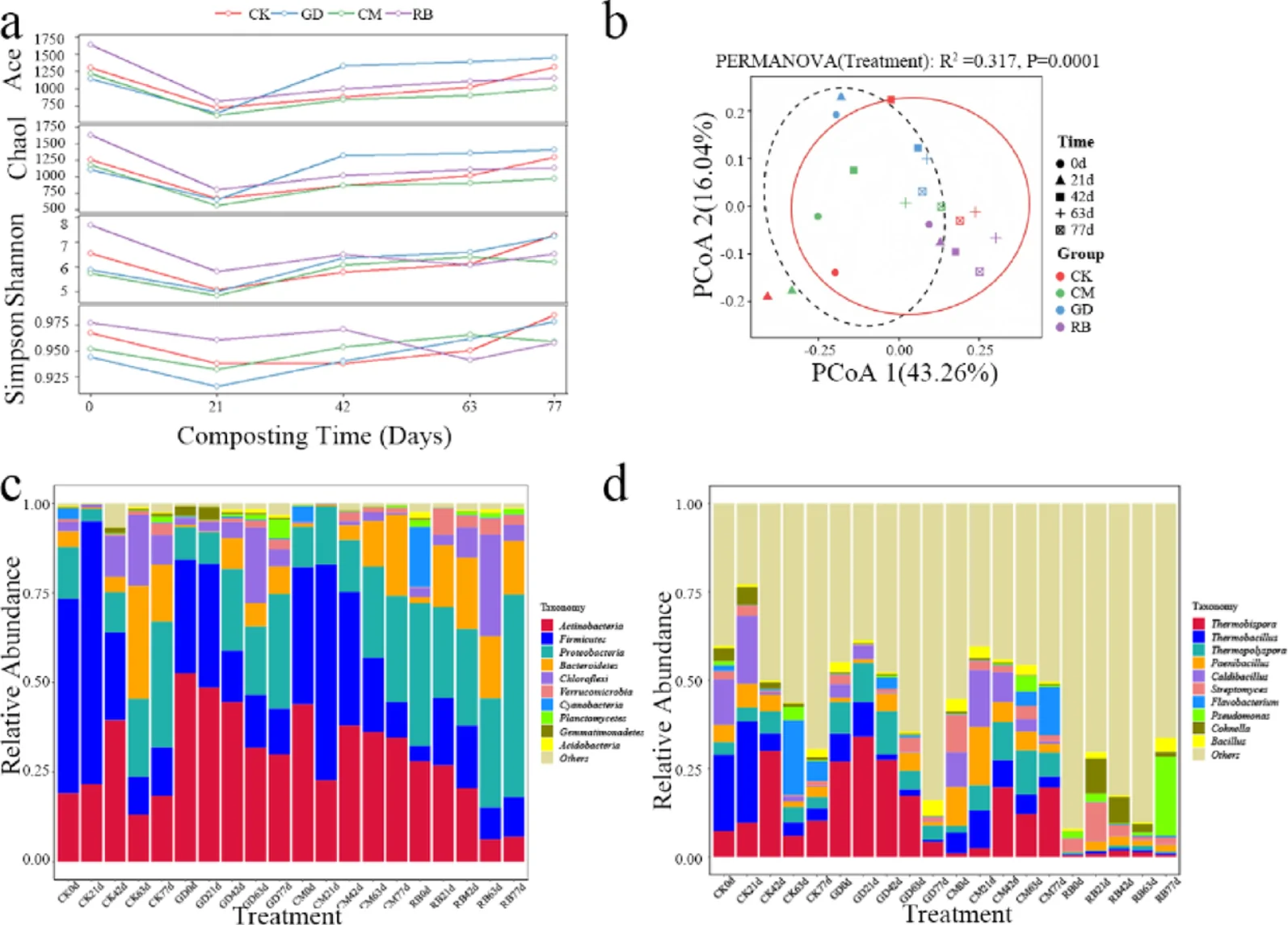
Bacterial alpha diversity, beta diversity, and community composition during composting. **A** Alpha diversity indices (Ace, Chao1, Shannon, and Simpson) of bacterial communities; **B** PCoA of bacterial community structure based on weighted UniFrac distances; **C** relative abundance of dominant bacterial phyla; and **D** relative abundance of dominant bacterial genera. *Note:* Only the top 10 taxa are displayed in (**C**) and (**D**), with the remaining taxa aggregated as “Others”. In (**B**), samples are color-coded according to treatment (CK, GD, CM, and RB) and shaped according to composting stage (D0, D21, D42, D63, and D77). Error bars indicate ± SE where applicable (*n* = 3). CK: *C. oleifera* shell + urea; CM: *C. oleifera* shell + urea + *C. oleifer*a seed meal; RB: *C. oleifera* shell + urea + rice bran; GD: *C. oleifera* shell + urea + goat dung. Abbreviations: PCoA: principal coordinate analysis

The temporal changes in bacterial alpha diversity are shown in Fig. 3a. At days 0 and 21, the RB treatment showed the highest observed Ace, Chao1, Shannon, and Simpson values among the four formulations. At day 42, GD showed the highest Ace and Chao1 values, whereas RB showed the highest Shannon and Simpson values. At day 63, GD had the highest Ace, Chao1, and Shannon values, whereas CM had the highest Simpson value. At day 77, GD showed the highest Ace and Chao1 values, whereas CK showed the highest Shannon and Simpson values. Overall, the four alpha-diversity indices exhibited treatment- and time-dependent fluctuations during composting. The PCoA of bacterial beta diversity showed that the first and second ordination axes explained 43.26% and 16.04% of the total variation, respectively, with a cumulative contribution of 59.3% (Fig. 3b). Restricted-permutation PERMANOVA indicated that bacterial composition was associated with both treatment (df = 3, pseudo-F = 3.486, R^2^ = 0.317, *P* = 0.0001) and time (df = 4, pseudo-F = 2.625, R^2^ = 0.319, *P* = 0.0001). Because each treatment-time cell had only one profile, these patterns were interpreted descriptively.

At the phylum level, the bacterial community was dominated by Actinobacteria, Firmicutes, Proteobacteria, Bacteroidetes, Chloroflexi, and Cyanobacteria, showing clear stage-specific succession. During the initial stage (0–21 d), Firmicutes predominated, accounting for 55–75% of the community, but its relative abundance declined continuously to 8–12% by day 77. In contrast, Proteobacteria, Bacteroidetes, and Chloroflexi increased markedly, with Chloroflexi reaching 16–22% at 77 d. Actinobacteria maintained a relatively high abundance throughout the composting period (10–52%) and peaked at GD group 0d (approximately 52%). The RB treatment followed a distinct successional trajectory in the later stage: Cyanobacteria increased significantly from 63 to 77 d (15–20%), whereas Actinobacteria decreased to the lowest level among all groups at 77 d (approximately 10%). Overall, the bacterial community shifted from a Firmicutes-dominated structure to a more diverse assemblage co-dominated by multiple phyla (Fig. 3c).

At the genus level, the thermophilic genera Thermobispora and Thermobacillus dominated during the thermophilic stage (0–42 d) (Fig. 3d). Thermobispora remained stable at 27–30% in the CK group, and Thermobacillus reached approximately 22% in the CK group at 0 d. Other heat-tolerant genera, such as Thermopolyspora and Paenibacillus, were also enriched at 21–42 d. During the cooling and maturation stage (63–77 d), these thermophilic genera declined sharply, with Thermobispora decreasing to 4–12%, whereas Streptomyces and Flavobacterium increased. The proportion of low-abundance taxa rose from 25 to 40% in the early stage to 60–78%, indicating a more diverse and even community. The successional trajectories differed among treatments. In the GD group, Pseudomonas was relatively abundant at 0 d (approximately 8%). In the RB group, Bacillus was the most abundant genus at 0 d (approximately 15%), Cohnella was markedly enriched at 21–42 d, and Pseudomonas increased substantially to approximately 22% at 77 d, becoming a signature genus at the end of maturation. These results indicate that composting time was the dominant factor driving the succession from thermophilic genera to a diversified maturation community, whereas the different amendments further shaped distinct genus-level patterns. However, due to a lack of replication, further verification is still required.

**Fig. 4:**
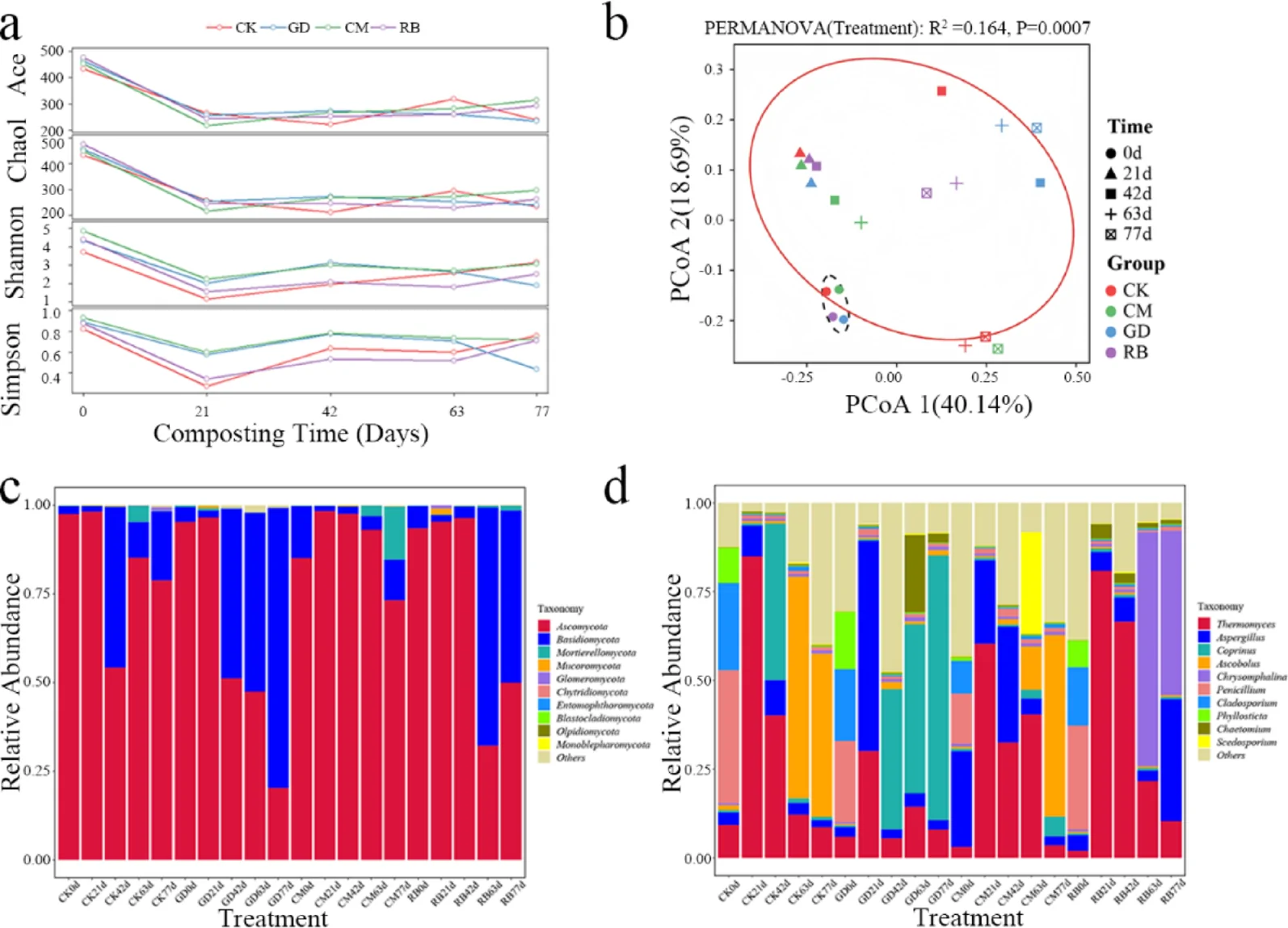
Fungal alpha diversity, beta diversity, and community composition during composting. **A** Alpha diversity indices (Ace, Chao1, Shannon, and Simpson) of fungal communities; **B** PCoA of fungal community structure based on weighted UniFrac distances; **C** Relative abundance of dominant fungal phyla; **D** Relative abundance of dominant fungal genera. *Note:* Only the top 10 taxa are displayed in (**C**) and (**D**), while the remaining taxa are grouped as “Others”. In (**B**), samples are color coded according to treatment (CK, GD, CM, and RB) and shaped according to composting stage (D0, D21, D42, D63, and D77). CK: *C. oleifera* shell + urea; CM: *C. oleifera* shell + urea + *C. oleifera* seed meal; RB: *C. oleifera* shell + urea + rice bran; GD: *C. oleifera* shell + urea + goat dung. Abbreviations: PCoA: principal coordinate analysis

The temporal changes in fungal alpha diversity are shown in Fig. 4a. Within each formulation, the Ace, Chao1, Shannon, and Simpson indices generally decreased markedly from day 0 to day 21, indicating a pronounced early decline in observed fungal richness and diversity. After day 21, the four indices fluctuated among formulations rather than showing a uniform recovery pattern. At day 77, CM showed the highest observed Ace and Chao1 values, whereas CK showed the highest Shannon and Simpson values; GD showed comparatively lower Shannon and Simpson values. Overall, fungal alpha diversity exhibited pronounced temporal variation and formulation-specific trajectories during composting. The PCoA of fungal beta diversity showed that the first and second axes explained 40.14% and 18.69% of the total variation, respectively (cumulative 58.83%, Fig. 4b). Fungal composition was also associated with treatment (df = 3, pseudo-F = 1.740, R^2^ = 0.164, *P* = 0.0007), but the temporal component explained a larger proportion of variation (df = 4, pseudo-F = 3.656, R^2^ = 0.459, *P* = 0.0001). With no independent profiles within treatment-time cells and no multivariate-dispersion assessment, these patterns do not establish replicated treatment effects or treatment × time interactions.

At the phylum level, Ascomycota dominated throughout the composting period (25–99%), followed by Basidiomycota (Fig. 4c). During the initial stage (0–21 d), nearly all samples were dominated by Ascomycota alone (> 88%). In the middle stage (42–63 d), Basidiomycota expanded markedly, reaching approximately 72%, 47%, and 45% in CM42d, GD42d, and CK63d, respectively, indicating a clear phylum-level shift. By 77 d, Ascomycota regained dominance (72% to 98%). Differences among treatments were mainly reflected in the expansion magnitude of Basidiomycota (CM group > GD group > CK group > RB group), suggesting that the amendments differentially regulated the phylum-level structure of the fungal community during the middle stage.

At the genus level, the fungal community showed a successional pattern characterized by Thermomyces dominance, mid-stage diversification, and treatment-specific divergence at the later stage (Fig. 4d). During the initial stage (0–21 d), the thermophilic genus Thermomyces was the most abundant, accounting for approximately 85% at CK0d and 40–60% in the other samples, followed by Aspergillus. In the middle stage (21–63 d), the community became more diverse: saprotrophic and decomposer genera such as Coprinus, Ascobolus, Cladosporium, and Chaetomium were enriched in different treatments (for example, Chaetomium reached approximately 55% at GD63d), forming a co-dominant assemblage together with Thermomyces and Aspergillus. At 77 d, the genus-level structure was clearly treatment-specific: Scedosporium increased to approximately 15% in the CK group, Thermomyces rebounded to approximately 65% in the GD group, and Penicillium expanded substantially to approximately 70% in the RB group, becoming the absolutely dominant genus at the end of maturation. These results indicate that Thermomyces was the core genus during the thermophilic stage, whereas saprotrophic fungi with potential cellulose- and lignin-degrading capacity took over during cooling and maturation, and the different amendments further selected distinct post-maturation fungal communities by modifying the microenvironment.

### Functional prediction

**Fig. 5:**
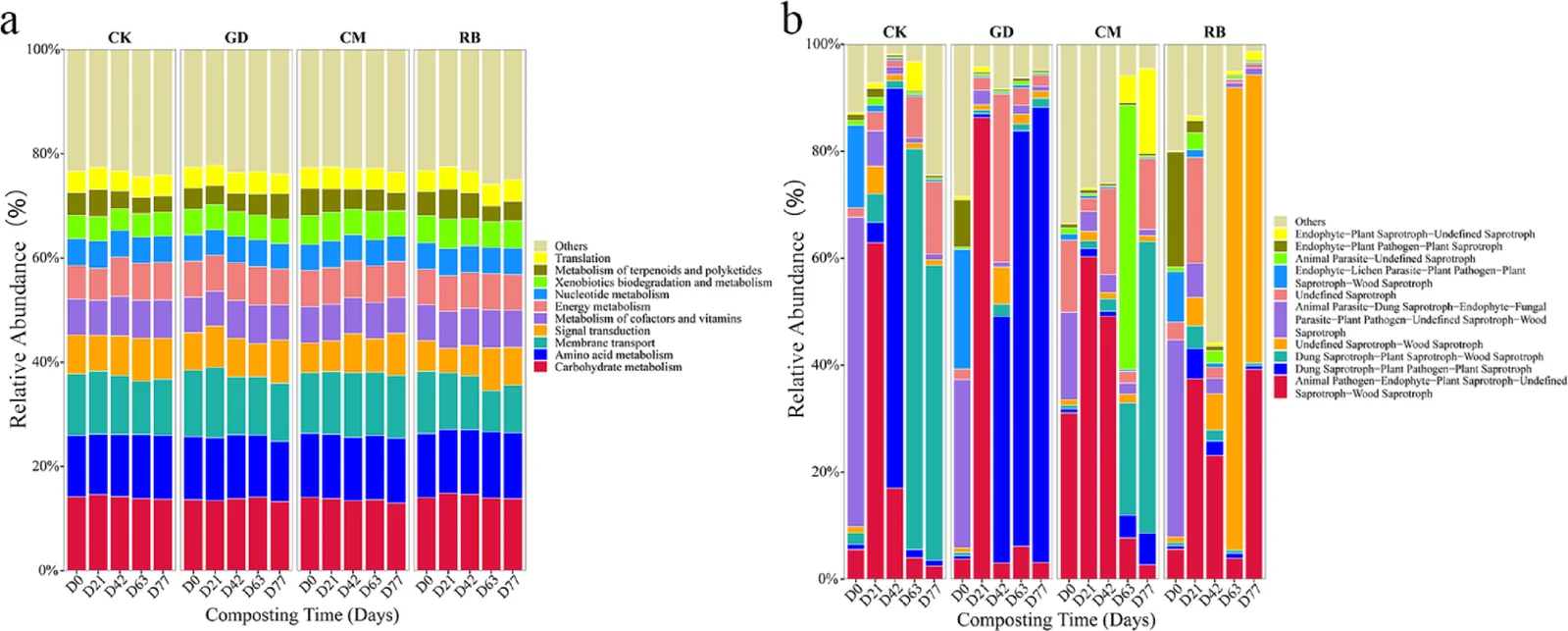
Predicted functional profiles of microbial communities during composting: **A** bacterial functional profiles predicted using Tax4Fun based on KEGG pathways; **B** fungal guilds inferred using FUNGuild. *Note:* Relative abundances of fungal guilds are displayed across different treatments and composting stages. Guild assignments represent inferred ecological functions based on ITS-derived taxonomic profiles. CK: *C. oleifera* shell + urea; CM: *C. oleifera* shell + urea + *C. oleifera* seed meal; RB: *C. oleifera* shell + urea + rice bran; GD: *C. oleifera* shell + urea + goat dung

To extend the community-level findings to the functional level, we predicted the functions of the fungal and bacterial communities. The results showed that treatment-driven bacterial turnover did not lead to divergence in overall functional potential (Fig. 5a). The fungal functional guilds showed clearly different successional trajectories between the control (CK group) and the three amended treatments (GD, CM, RB group). CK group underwent a gradual natural succession. Endophytic fungi and plant pathogen-associated guilds dominated in the early stage, followed by a brief transition through the Animal Parasite-Dung Saprotroph guild in the middle stage. Saprotrophic guilds became dominant only in the late-stage (days 63 to 77). In contrast, the shift toward saprotrophy occurred earlier in all three amended treatments, although through different pathways. In GD, the relative abundance of the Dung Saprotroph–Plant Pathogen–Plant Saprotroph guild increased from 0.74% at day 21 to 46.10% at day 42, 77.68% at day 63, and 85.11% at day 77, showing the fastest and most complete transition. In the CM group, the Animal Parasite-Undefined Saprotroph and Dung Saprotroph-Plant Saprotroph-Wood Saprotroph guilds alternated substantially at days 42–63, indicating the most intensive community reorganization. In RB, the Dung Saprotroph-Plant Saprotroph-Wood Saprotroph guild showed a pulse-like bloom (approximately 80%) at day 42 and then declined. These are relative abundances among retained guild-annotated reads, not measured functional rates. Composite categories containing both pathogen and saprotroph labels cannot establish a transition to an exclusively saprotrophic community, pathogen elimination, or product safety. With one pooled profile per treatment-time cell, these patterns remain descriptive.

### Microbe–environment relationships

**Fig. 6:**
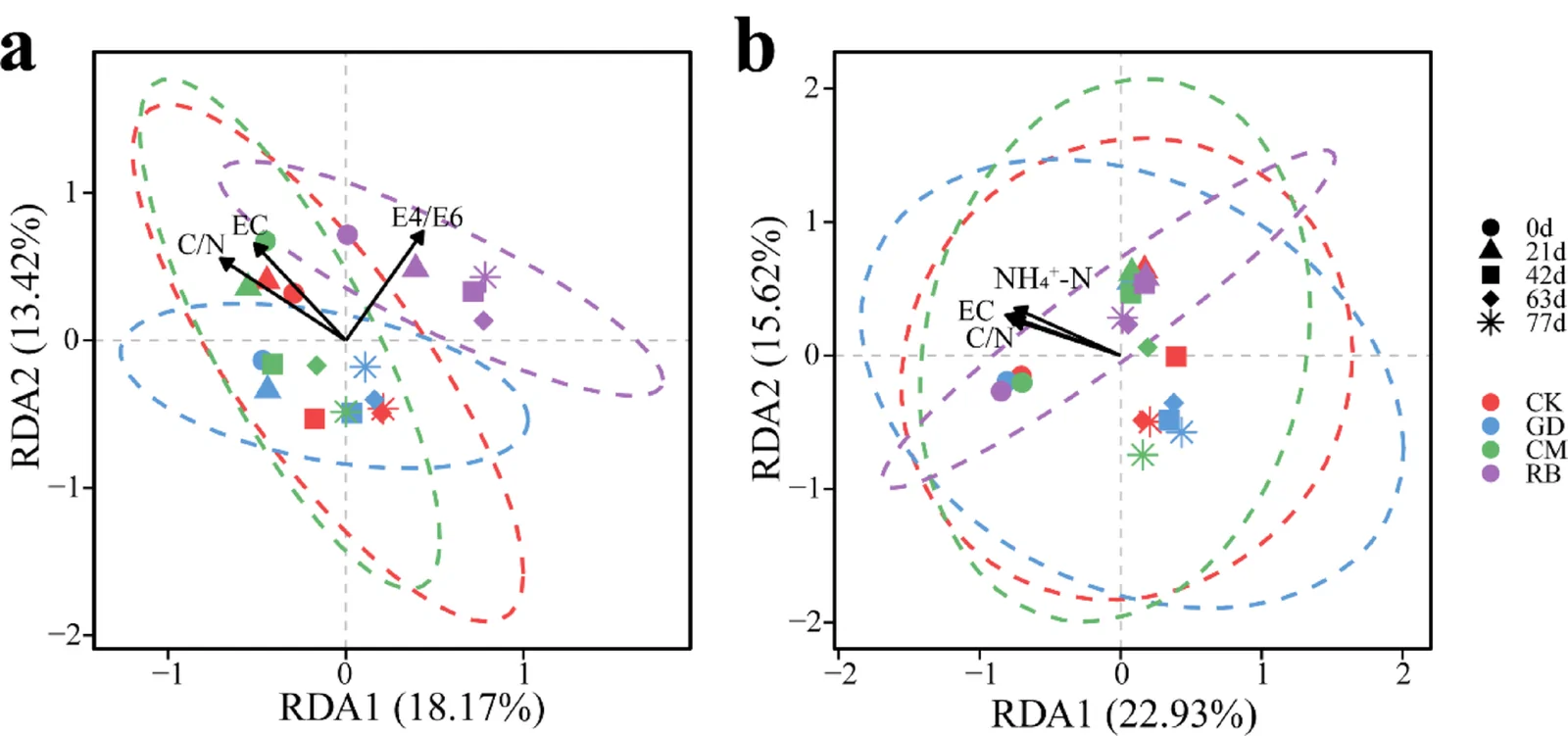
RDA of bacterial and fungal community composition constrained by physicochemical properties during composting. **A** Bacterial communities based on Hellinger–transformed bacterial OTU abundance matrices. **B** Fungal communities based on Hellinger–transformed fungal OTU abundance matrices. *Note:* Points represent samples and are colored according to treatment, with point shapes indicating composting stages. Arrows indicate the three physicochemical variables most strongly associated with the ordination structure. The length of the arrows reflects the strength of association with the ordination axes, while the direction of the arrows indicates increasing values of each variable. CK: *C. oleifera* shell + urea; CM: *C. oleifera* shell + urea + *C. oleifera* seed meal; RB: *C. oleifera* shell + urea + rice bran; GD: *C. oleifera* shell + urea + goat dung. Abbreviations: C/N: carbon/nitrogen; E4/E6: ratio of absorbance at 465 nm to that at 665 nm in the same bicarbonate compost extract; EC: electrical conductivity

RDA showed that physicochemical factors explained different proportions of variation in the bacterial and fungal communities. For the Hellinger-transformed bacterial OTU matrix, RDA1 and RDA2 explained 18.17% and 13.42% of the community variation, respectively (31.59% in total). The factors most strongly associated with the ordination structure were C/N, EC, and E4/E6. C/N and EC pointed toward the upper left of the ordination space, whereas E4/E6 pointed toward the upper right, indicating associations of the measured C/N ratio, EC, and optical index with bacterial community composition (Fig. 6a). For the fungal OTU matrix, RDA1 and RDA2 explained 22.93% and 15.62% of the variation, respectively (38.55% in total). The variables most strongly associated with fungal composition were NH_4_^+^–N, EC, and C/N, which pointed in highly consistent directions and together formed a physicochemical gradient that decreased with composting progress (Fig. 6b). Samples of both communities shifted regularly along the corresponding gradients with composting stage. Early samples (0 and 21 d) clustered on the side with high C/N and high EC (and high NH_4_^+^–N for the fungal community) and gradually moved in the opposite direction as composting proceeded. These ordination patterns describe covariation and do not identify a causal transformation pathway. Notably, the spatial separation patterns between CK and the amended treatments differed clearly between the two communities. For bacteria, CK samples were mainly distributed in the lower-left region of the ordination space, associated with high C/N and high EC, whereas samples of GD, CM, and RB shifted toward decreasing C/N and EC, with the largest shift observed in RB. This may suggest that the bacterial succession in the three amended treatments was associated with faster carbon and nitrogen transformation. However, microbial contributions were not measured. In contrast, fungal samples clustered mainly by composting stage rather than by treatment. Samples of the same stage from different treatments were close to each other, and the plotted ellipses of the four treatments overlapped extensively, with no clear separation between CK and the amended treatments. This suggests that temporal covariation in the sampled fungal profiles, without quantifying independent treatment effects. Ellipses summarize the distribution of time points along each treatment trajectory and do not represent uncertainty among replicate bins.

**Fig. 7:**
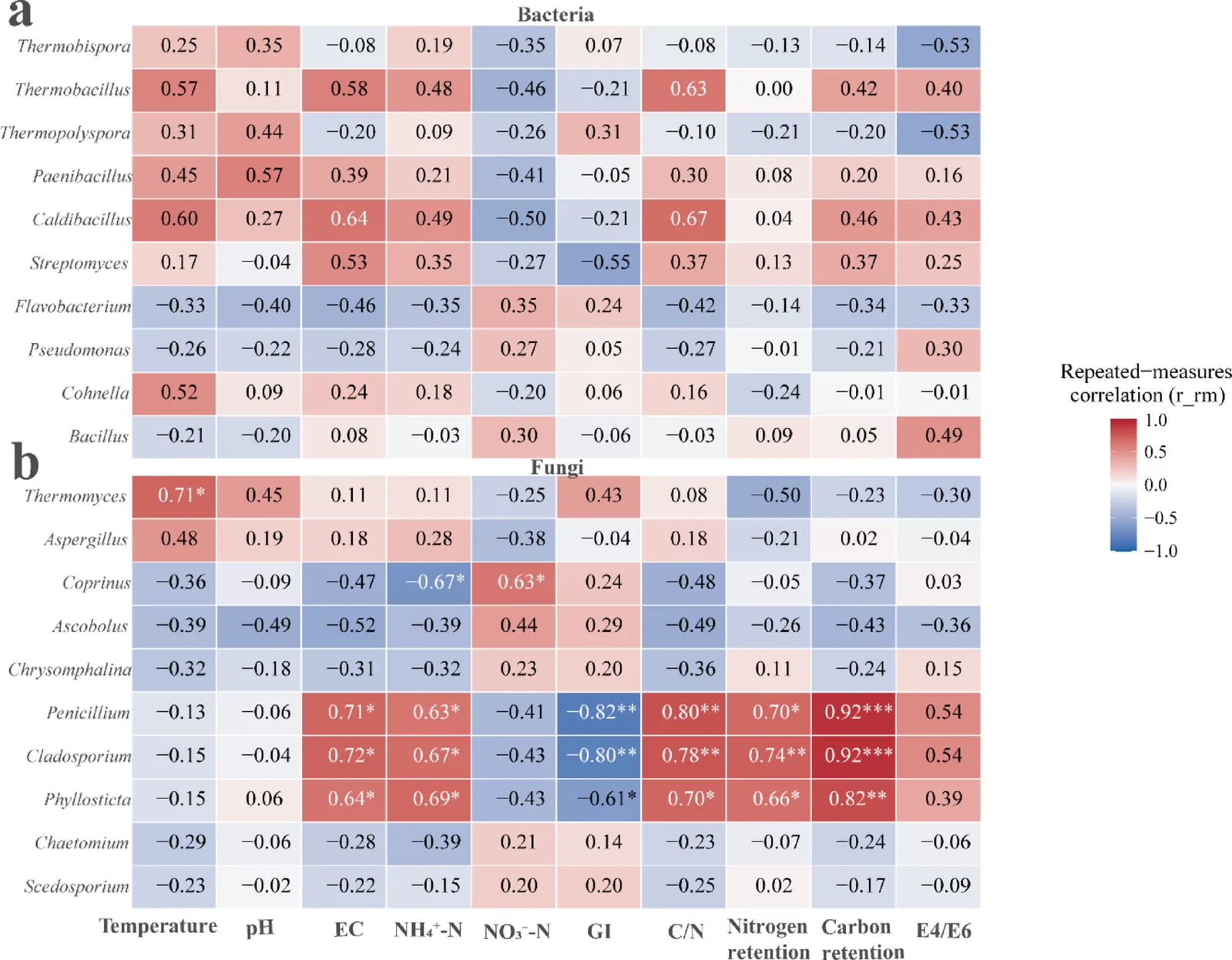
Repeated-measures correlations between dominant microbial genera and physicochemical properties during composting. **A** Bacterial genera; **B** fungal genera. Correlations were calculated from 20 treatment-by-time profiles representing four treatment trajectories across five sampling times; physicochemical triplicates were averaged within each treatment-time combination before matching to the microbial profile (df = 15). Cell values are repeated-measures correlation coefficients (r_rm). Benjamini–Hochberg correction was applied separately to the 100 comparisons in each kingdom. *, q < 0.05; **, q < 0.01; ***, q < 0.001. Abbreviations: C/N: carbon/nitrogen; E4/E6: ratio of absorbance at 465 nm to that at 665 nm in the same bicarbonate compost extract; EC: electrical conductivity

After accounting for the repeated treatment trajectories and controlling the false discovery rate, none of the 100 bacterial genus–environment associations remained significant (all q ≥ 0.262, Fig. 7a). In contrast, 21 of the 100 fungal associations remained significant at q < 0.05. The strongest positive associations involved carbon retention with Cladosporium (r_rm = 0.924, q = 6.11 × 10^− 6^) and Penicillium (r_rm = 0.924, q = 6.11 × 10^− 6^), whereas GI was negatively associated with Penicillium (r_rm = − 0.842, q = 0.000733) and Cladosporium (r_rm = − 0.823, q = 0.00106) (Fig. 7b). Grouping by treatment does not remove shared temporal trends or guarantee independent residuals; the associations therefore describe exploratory covariation and do not establish microbial mechanisms.

## Discussion

*C. oleifera* shells have a high C/N ratio and lignin content, which can hinder composting when the shells are used alone. Exogenous nitrogen is therefore commonly added to facilitate microbial growth and substrate conversion. In the present study, we compared a urea-only formulation with three formulations in which urea was combined with GD, CM, or RB. All treatments were adjusted to a common initial C/N ratio and subjected to the same moisture, inoculation, and turning regime. This common C/N ratio established a comparable initial stoichiometric condition, but it did not eliminate differences among the co-substrates in labile-carbon supply, mineral composition, indigenous microorganisms, potential inhibitory compounds, or physical properties. Accordingly, our experiment evaluates the integrated effects of the tested composting formulations rather than the isolated effect of nitrogen form or nitrogen-release synchrony. Within this scope, the formulations showed distinct patterns of temperature development, carbon and nitrogen retention, inorganic nitrogen dynamics, compost maturity, and microbial succession. The pooled microbial profiles suggested different trajectories among formulations. Together, these findings suggest that differences in co-substrates composition and properties contributed to the observed composting outcomes.

Temperature dynamics are an integrated outcome of microbial decomposition and heat transfer within the compost pile, both of which are influenced by feedstock composition and pile properties (Finstein and Morris 1975; de Bertoldi et al. 1983; Mason 2006). Relative to the delayed and short thermophilic profile of the urea-only formulation, the three co-substrate-amended formulations exhibited three distinct thermal responses: rapid temperature development in RB, the highest peak temperature in CM, and the longest cumulative thermophilic duration in GD. Those differences indicate that a common initial C/N ratio did not create equivalent decomposition environments among the treatments; instead, the co-substrates were associated with different combinations of substrate quality and pile characteristics that influenced the rate and persistence of thermophilic activity. The thermal phase is important because it establishes the physicochemical context for the subsequent differences in organic-matter transformation, nitrogen dynamics, and microbial succession. Similar formulation-dependent thermal responses have been reported in composting systems with different feedstock compositions (Zhu 2007; Liu et al. 2025). In addition, Turning may have introduced cooler ambient air into the compost pile, causing a transient decrease in temperature. The improved aeration following turning could then promote microbial growth and organic matter decomposition, increasing heat production and allowing the compost temperature to rise again. Repeated cooling and reheating following weekly turning may therefore have contributed to the multiple temperature peaks observed in temperature curve.

Differences in carbon availability and feedstock composition may contribute to the observed divergence in nitrogen retention. The fate of mineralized nitrogen depends on the competition between ammonia volatilization and microbial assimilation, and microbial assimilation depends on available carbon and an active microbial community (Shan et al. 2021; Hoang et al. 2022). RB contains approximately 50% carbohydrate, 15–20% fat, and 12–15% protein (Gul et al. 2015; Sapwarobol et al. 2021). This nutrient combination could support microbial nitrogen assimilation, but microbial biomass nitrogen was not measured. Its nitrogen retention rate (91.64 ± 0.96%) was higher than that of the other treatments; neither assimilation nor nitrogen fixation can be quantified from these measurements. GD group supplied less readily degradable carbon than RB group, and its nitrogen retention was intermediate (72.71 ± 1.18%). The most notable case was CM. Although *C. oleifera* seed meal is not low in nitrogen, its nitrogen retention (66.97 ± 1.65%) was almost identical to that of CK group (65.65 ± 1.83%), probably because the meal is rich in tea saponin and tannins. Tea saponin has been shown to possess broad-spectrum antimicrobial activity (Zhao et al. 2020). Whether saponins affected microbial assimilation or nitrogen volatilization in this treatment remains untested. This result suggests that recycling homologous materials may entail biochemical inhibition, and that the choice of an organic nitrogen source cannot be based on nutrient content alone. CK had the lowest estimated carbon retention (28.56 ± 1.36%); a priming effect and its consequences for nitrogen immobilization were not directly tested.

The measured inorganic nitrogen pools provide further descriptive evidence of treatment differences. At the end of composting, GD group had the highest NO_3_^−^–N (142.63 mg/kg), showing the highest measured nitrate concentration. The sources and rates of nitrate formation cannot be determined without direct measurements of nitrifiers or nitrification activity. RB showed the opposite pattern, with the highest NH_4_^+^–N (777.92 mg/kg) but relatively low NO_3_^−^–N (53.45 mg/kg) at the end. Its high estimated total nitrogen retention cannot establish the amount stored in microbial or other organic forms, or the magnitude of volatilization. GD had the highest final total nutrient concentration (5.20%), which may partly reflect differences in initial P and K inputs, particularly the high K content of goat dung, and the concentration effect associated with organic matter degradation and dry-matter loss. Because nutrient concentrations were expressed on a dry-matter basis, higher concentrations do not necessarily indicate greater nutrient conservation. Nitrogen retention was estimated using mass-balance calculations, whereas P and K retention were not quantified. Therefore, GD’s higher total nutrient concentration does not establish enhanced overall nutrient conservation. Although RB had the highest estimated nitrogen retention, its final total nutrient content (3.66%) was below the 4.0% minimum specified in NY/T 525–2021 (Ministry of Agriculture and Rural Affairs of the People’s Republic of China 2021). Thus, high nitrogen retention alone does not ensure that the final compost meets the cited total-nutrient criterion. Practical assessment must also consider temperature history, C/N, GI, pH, EC, nutrient composition, and intended use. These indicators address different aspects of stabilization, phytotoxicity, and nutrient value; they do not substitute for final-product contaminant and hygiene testing. No formulation can be recommended as a verified safe agricultural product from these data alone.

The successional pattern of the fungal community was found. Ascomycetes dominated early composting (relative abundance above 88%, approximately 85% at CK0d), Thermotolerant taxa such as Thermomyces may have persisted during the thermophilic phase, but the substrates supporting their growth were not measured. Basidiomycetes increased during in the middle stage (42–63 d; about 72% at CM42d and 47% at GD42d). Cooling and changes in substrate availability may have favored this shift, but lignin degradation was not measured directly. Ascomycetes resumed dominance during the final maturation stage. The mid-stage increase in Basidiomycota is consistent with changing and substrate conditions, although their separate contributions cannot be resolved. Bacterial genera also varied over time and among formulations. During the thermophilic phase, Thermobispora (maintained at 27–30% in CK from 0 to 42 d) and Thermobacillus were prominent; after cooling, Streptomyces and Flavobacterium became more abundant. This succession is broadly consistent with patterns reported in cattle manure composting (Wang et al. 2018). In the late-stage of RB group, Pseudomonas was enriched to about 22%, possibly reflecting substrate availability, although neither its functional activity nor the persistence of readily degradable RB fractions was measured. The increase in Cyanobacteria to 15–20% in RB composting warrants verification its origin and function remain uncertain. These genus-level differences suggest formulation-associated variation in the maturation community, even where broader community comparisons were not significant.

Predicted KEGG bacterial functional profiles were similar across treatments and were dominated by carbohydrate metabolism, amino acid metabolism, and membrane transport. This pattern may reflect functional redundancy across bacterial taxa, but predicted pathways do not demonstrate equivalent in situ activity (Langille et al. 2013; Louca et al. 2018). Fungal taxa can differ in extracellular enzyme repertoires and substrate use, potentially yielding more variable predicted guild profiles. Consistent with this possibility, FUNGuild assignments varied among formulations (Nguyen et al. 2016): saprotrophic guilds already accounted for 60–85% in GD group from D21 onward, potentially reflecting the manure amendment, although the origin and activity of these fungi were not established; RB showed a pulsed bloom of saprotrophic guilds at D42 (about 80%), which may relate to changes in RB-derived substrates; CM group underwent drastic reorganization from D42 to D63, but the proposed roles of substrate degradation and saponins remain speculative; whereas CK showed a gradual shift in predicted guild composition from endophytic or pathogenic assignments toward saprotrophic assignments. FUNGuild labels are ecological predictions and do not establish measured fungal activity.

Redundancy analysis identified physicochemical associations with community composition. The bacterial community was associated mainly with C/N, EC and E4/E6, whereas the fungal community was associated mainly with NH_4_^+^-N, EC and C/N, these associations do not establish distinct functional roles. CK samples were consistently distributed in the high EC and high C/N region, in association with higher EC and C/N. In contrast, GD, CM, and RB samples shifted along the direction of increasing E4/E6 and decreasing C/N, with RB showing the most pronounced shift in the bacterial ordination. These patterns indicate compositional covariation with measured compost properties. In the Fig. 7 analysis, no bacterial genus–environment association survived false-discovery-rate correction (all q ≥ 0.262); 21 fungal associations did, including positive associations of carbon retention with Cladosporium and Penicillium (both r_rm = 0.924, q = 6.11 × 10^− 6^). These associations are exploratory because there were only four pooled trajectories, common time trends were not removed, and serial dependence could not be excluded. Thus, neither bacterial nor fungal succession can be assigned a specific role in NH_3_ emission or nitrogen conservation from these correlations.

Several limitations of this study should be acknowledged. First, each treatment comprised only three biological replicates (compost bins), and the amplicon sequencing dataset provided one pooled microbial profile per treatment-time combination rather than three independent profiles. Restricted permutations do not restore this replication, and multivariate dispersion was not assessed. Microbial patterns and correlations were therefore treated as exploratory. Second, OTU clustering at 97% similarity was used rather than amplicon sequence variant methods, which can improve sequence resolution. Third, the experiment was conducted at a single location over a single 77-day composting period, so generalization to other climates, feedstocks, or seasons requires caution. Fourth, because the organic amendments differed in carbon, nitrogen, and lignin content in addition to nitrogen form, the effects of amendment composition and nitrogen source could not be fully separated. Fifth, NH_3_, N_2_O, N_2_, microbial biomass nitrogen, functional genes, and enzyme activities were not measured; mass-balance estimates and inorganic nitrogen pools cannot identify individual nitrogen transformation or loss pathways. Sixth, heavy-metal measurements were limited to the initial *C. oleifera* shell. Potential contaminants introduced by goat dung, *C. oleifera* seed meal, rice bran, and other inputs were not comprehensively assessed, and final-product contaminant measurements were unavailable. Therefore, agricultural-use safety cannot be established from the shell measurements alone and requires verification through final-product testing.

## Conclusion

Under the tested formulations and a common initial C/N ratio, combining urea with goat dung, *C. oleifera* seed meal, or rice bran resulted in distinct composting trajectories compared with urea alone. The rice-bran formulation achieved the highest estimated nitrogen retention, whereas the goat-dung formulation yielded the highest final total nutrient concentration. RB nevertheless remained below the cited 4.0% total-nutrient criterion. Microbial profiles and inferred functions provide exploratory associations rather than demonstrated metabolic mechanisms. These findings highlight the importance of amendment selection in balancing nitrogen retention and final compost nutrient content during *C. oleifera* shell composting. Further evaluation of overall nutrient conservation and final-product safety is needed before agricultural application.

Table.

## Supplementary Information

Below is the link to the electronic supplementary material.

Supplementary Material 1

Supplementary Material 2

## Data Availability

The bacterial 16 S rRNA gene and fungal ITS amplicon-sequencing datasets generated from Camellia oleifera shell compost samples in this study have been deposited in the NCBI Sequence Read Archive under BioProject accession PRJNA1537769 and SRA Study accession SRP741655. The BioSample accessions are SAMN63706898–SAMN63706917, and the SRA Run accessions are SRR40963347–SRR40963386. Additional data supporting the findings of this study are available from the corresponding author upon reasonable request.

## Abbreviations

16S rRNA: 16 S ribosomal RNA
Ace: Abundance-based coverage estimator
BLAST: Basic Local Alignment Search Tool
C/N: Carbon-to-nitrogen ratio
CFU: Colony-forming unit
CK: Urea-only control
CM: Camellia oleifera seed meal
EC: Electrical conductivity
E4/E6: Ratio of absorbance at 465 nm to that at 665 nm in the same compost extract
GD: Goat dung
GI: Germination index
HSD: Honestly significant difference
ITS: Internal transcribed spacer
KEGG: Kyoto Encyclopedia of Genes and Genomes
NCBI: National Center for Biotechnology Information
NA: Not applicable
NH_4_^+^–N: Ammonium nitrogen
NO_3_^−^–N: Nitrate nitrogen
OTU: Operational taxonomic unit
PCoA: Principal coordinate analysis
PCR: Polymerase chain reaction
PERMANOVA: Permutational multivariate analysis of variance
QIIME: Quantitative Insights Into Microbial Ecology
RB: Rice bran
RDA: Redundancy analysis
SD: Standard deviation
SE: Standard error
TC: Total carbon
TK: Total potassium
TN: Total nitrogen
TP: Total phosphorus
UPGMA: Unweighted pair group method with arithmetic mean
