# Supplementary material for: Composting performance, nitrogen retention, and microbial succession differ among urea-only and organic co-substrate-amended Camellia oleifera shell formulations
Source: Bioresour Bioprocess. 2026 Nov 12;13(1):165. doi: 10.1186/s40643-026-01161-x (PMC13759932; doi:10.1186/s40643-026-01161-x)
Supplement: Supplementary file 1 [file 40643_2026_1161_MOESM1_ESM.docx]

Table S2. Mass of each component in each pile

| **Treatment** | **C. oleifera shell wet/dry weight (kg bin⁻¹)** | **Organic co-substrate wet/dry weight (kg bin⁻¹)** | **Urea (kg bin⁻¹)** | **Shell/co-substrate dry-weight ratio** | **Activated microbial inoculum solution (mL bin⁻¹)** |
| --- | --- | --- | --- | --- | --- |
| CK | 48.843/41.516 | None | 1.089 | — | 1000 |
| GD | 39.766/33.801 | Goat dung: 9.495/8.450 | 0.598 | 4:1 | 1000 |
| CM | 39.204/33.323 | C. oleifera meal: 9.801/8.331 | 0.942 | 4:1 | 1000 |
| RB | 40.230/34.195 | Rice bran: 9.095/8.549 | 0.622 | 4:1 | 1000 |

Abbreviations: CK: *C. oleifera* shell + urea; CM: *C. oleifera* shell + urea + *C. oleifera* seed meal; RB: *C. oleifer*a shell + urea + rice bran; GD: *C. oleifera* shell + urea + goat dung.
